# TCRmodel2: high-resolution modeling of T cell receptor recognition using deep learning

**DOI:** 10.1093/nar/gkad356

**Published:** 2023-05-04

**Authors:** Rui Yin, Helder V Ribeiro-Filho, Valerie Lin, Ragul Gowthaman, Melyssa Cheung, Brian G Pierce

**Affiliations:** University of Maryland Institute for Bioscience and Biotechnology Research, Rockville, MD 20850, USA; Department of Cell Biology and Molecular Genetics, University of Maryland, College Park, MD 20742, USA; University of Maryland Institute for Bioscience and Biotechnology Research, Rockville, MD 20850, USA; Brazilian Biosciences National Laboratory, Brazilian Center for Research in Energy and Materials, Campinas 13083-100, Brazil; University of Maryland Institute for Bioscience and Biotechnology Research, Rockville, MD 20850, USA; Thomas S. Wootton High School, Rockville, MD 20850, USA; University of Maryland Institute for Bioscience and Biotechnology Research, Rockville, MD 20850, USA; Department of Cell Biology and Molecular Genetics, University of Maryland, College Park, MD 20742, USA; University of Maryland Institute for Bioscience and Biotechnology Research, Rockville, MD 20850, USA; Department of Chemistry and Biochemistry, University of Maryland, College Park, MD 20742, USA; University of Maryland Institute for Bioscience and Biotechnology Research, Rockville, MD 20850, USA; Department of Cell Biology and Molecular Genetics, University of Maryland, College Park, MD 20742, USA; University of Maryland Marlene and Stewart Greenebaum Comprehensive Cancer Center, Baltimore, MD 21201, USA

## Abstract

The cellular immune system, which is a critical component of human immunity, uses T cell receptors (TCRs) to recognize antigenic proteins in the form of peptides presented by major histocompatibility complex (MHC) proteins. Accurate definition of the structural basis of TCRs and their engagement of peptide–MHCs can provide major insights into normal and aberrant immunity, and can help guide the design of vaccines and immunotherapeutics. Given the limited amount of experimentally determined TCR–peptide–MHC structures and the vast amount of TCRs within each individual as well as antigenic targets, accurate computational modeling approaches are needed. Here, we report a major update to our web server, TCRmodel, which was originally developed to model unbound TCRs from sequence, to now model TCR–peptide–MHC complexes from sequence, utilizing several adaptations of AlphaFold. This method, named TCRmodel2, allows users to submit sequences through an easy-to-use interface and shows similar or greater accuracy than AlphaFold and other methods to model TCR–peptide–MHC complexes based on benchmarking. It can generate models of complexes in 15 minutes, and output models are provided with confidence scores and an integrated molecular viewer. TCRmodel2 is available at https://tcrmodel.ibbr.umd.edu.

## Graphical Abstract

**Graphical Abstract:**
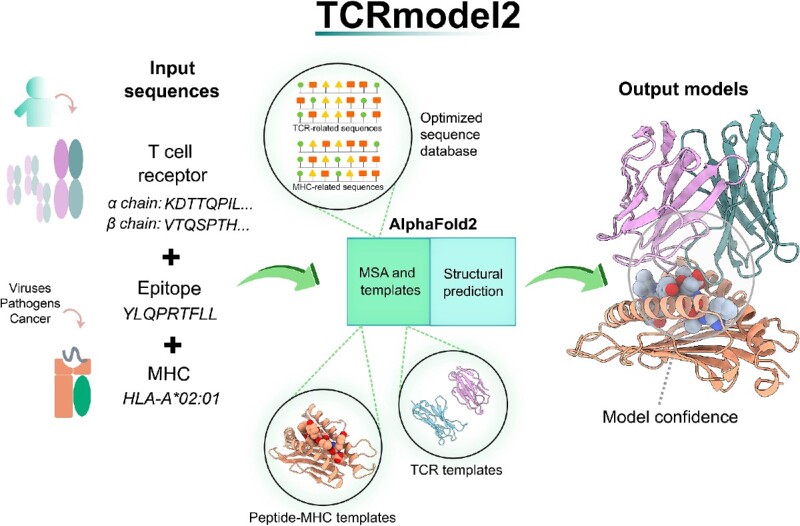
The workflow and key elements of TCRmodel2.

## INTRODUCTION

T cell immunity is a key component of immune protection from viruses and pathogens (1), such as SARS-CoV-2 (2). Additionally, T cells and T cell receptors (TCRs) often play a role in autoimmunity (3,4), and TCRs are increasingly being utilized as therapeutics in clinical and preclinical studies (5–7). Understanding the structural basis of TCR recognition of peptide–major histocompatibility complex (pMHC) targets can yield major mechanistic insights (3,4,8,9) and provide the means to perform structure-based design of TCR specificity or affinity (10,11). While several hundred high-resolution structures of TCR–pMHC complexes have been determined experimentally and are available in the Protein Data Bank (PDB) (12), this represents only a small fraction of TCRs [with millions of TCRs in each human repertoire (13)], and high-throughput sequencing and screening technologies are enabling large sets of antigen-specific TCR sequences to be routinely identified (14). The capability to perform accurate computational modeling of TCRs and TCR–pMHC complex structures would be highly useful, effectively bridging the gap between TCR sequence and 3D structural information. Such algorithms and models could be used for structure-based TCR design, or generalizable prediction of ‘unseen’ TCR epitopes, which represents a major challenge in computational biology (15) that may potentially be addressed through structure-based methods (16,17).

Several algorithms have been developed to perform modeling of unbound TCRs (18–20), and TCR–pMHC complexes (21–23) from sequence or unbound structures, primarily through template-based modeling combined with energy minimization. These approaches can often be unsuccessful due to limitations of templates coupled with the flexibility and diversity of TCR complementarity-determining region (CDR) loops, and the wide range of TCR–pMHC docking orientations. Recently, deep learning-based structure prediction methods, and particularly AlphaFold (24), have proven remarkably successful in predicting structures of monomeric proteins (24) and multimeric proteins (25) from sequence. While our own initial benchmarking of AlphaFold for modeling TCR–pMHC complexes showed limited success (2 out of 14 cases with near-native model accuracy) (26), its success in some cases showed that it is possible in principle to ‘fold and dock’ TCR–pMHC complexes with deep learning, and a recent study demonstrated that AlphaFold can be fine-tuned and optimized to model TCR–pMHC complexes (16).

Here, we describe the development of TCRmodel2, which is a major update of our previously released TCR modeling web server, TCRmodel (18). While the previous version used template-based modeling and Rosetta (27) to generate unbound TCR structural models from sequence, TCRmodel2 uses AlphaFold to generate models of TCR–pMHC complexes, with several modifications to improve its speed and accuracy. TCRmodel2 can also generate models of unbound TCRs using the same AlphaFold-based framework. Based on benchmarking, TCRmodel2 generates models of TCR–pMHC complexes with greater accuracy than AlphaFold and previously developed TCR–pMHC modeling methods, and it is over 10 times faster than the default AlphaFold protocol. To enable progress in structural immunology, we provide TCRmodel2 to the community as a web server, with user-friendly features such as multiple sequence input options, interactive structural visualization and model confidence scores.

## MATERIALS AND METHODS

### TCRmodel2 algorithm

The TCRmodel2 modeling pipeline was generated through several modifications of the AlphaFold pipeline and database, as noted below. These changes were separately implemented in the AlphaFold v2.2 and v2.3 codes (both downloaded from the AlphaFold GitHub repository), to enable comparative performance of the two AlphaFold models in the context of TCRmodel2.

#### Multiple sequence alignment database

Given that the AlphaFold feature selection stage includes sequence searches against large databases containing a large variety of proteins for each input chain, we reduced the databases to contain prospective TCR and MHC hits to speed up the multiple sequence alignment (MSA) building step. The AlphaFold pipeline was run with four representative human and murine TCR and MHC sequences, using the reduced database option. All hits from the TCR and MHC sequence searches against the Small BFD [Big Fantastic Database (24)], UniRef90 and UniProt AlphaFold databases were combined into new database files in FASTA format, replacing the full database files. The resultant databases have sizes of 450 (Small BFD), 43 638 (UniRef90) and 145 999 (UniProt) sequences, with the sequences collectively comprising 52 096 TCR-related sequences and 137 991 MHC-related sequences.

#### TCR templates

The AlphaFold template search, which utilizes an MSA built from the input sequence to search against PDB sequences to identify templates for each input chain (24), was found to identify non-TCR immunoglobulin structures as templates for TCR chains, versus TCR chain structures with closer identity to the input sequence (e.g. a human TCR with the same germline gene). As this was due to the use of the MSA in the query against the PDB sequences (which is useful when distant orthologs may need to be detected as candidate templates), we modified AlphaFold to only utilize the input TCR sequences rather than MSAs to search against the PDB.

#### Peptide–MHC structural templates

AlphaFold was modified to utilize pMHC complex structures as input templates by representing peptide and MHC as a single structure, with a chain break between peptide and MHC given by a residue index shift as used by ColabFold (28). Template featurization of pMHC templates from PDB structures was conducted using the AlphaFold modification described by Motmaen *et al.* (29). To obtain pMHC templates, pMHC structures with resolution ≤3.5 Å were obtained from TCR3d. For Class II pMHC structures, peptides were trimmed to include the 9-mer core sequence plus one flanking residue at each terminus, and due to diversity of length and conformation of Class I peptides, additional unbound Class I pMHC structures within the resolution cutoff were identified from the PDB and included in the set. To account for peptide structural heterogeneity while limiting redundancy, up to two structures with identical pMHC sequences were retained. In total, our template set includes 884 Class I and 44 Class II pMHC template structures. At the peptide–MHC template selection stage, structures containing peptides with the same length as query sequence are identified, and ranked first by MHC similarity score and then by peptide similarity score if multiple identical MHCs are identified. Similarity scores for MHC and peptide sequences are calculated using BLOSUM62 in the Bio.Align Biopython package (30), with a large gap penalty (−100) for peptide alignments to ensure ungapped peptide alignments and template scoring.

#### Model scoring

For TCRmodel2 models, we provide AlphaFold-generated confidence scores, specifically the average predicted local difference distance test (pLDDT, corresponding to local structural accuracy), predicted TM (pTM, corresponding to overall topological accuracy) score, ipTM (pTM calculated for interchain interfaces) score and model confidence, which is a linear combination of pTM and ipTM (0.2 × pTM + 0.8 × ipTM) (25). Additionally, for TCR–pMHC complex models we modified AlphaFold to calculate TCR–pMHC ipTM, which corresponds to the interface pTM score calculated only across the interface between TCR and pMHC, versus the default ipTM, which is calculated between all chains (e.g. peptide–MHC, TCR α and β chains). The TCR–pMHC ipTM score is calculated by modifying the chain IDs of the TCR–pMHC complex predictions in AlphaFold at the time of ipTM calculation to represent the TCR and pMHC each as one chain. TCRmodel2 also calculates average pLDDT score for each of the CDR3 loops, to enable users to specifically view the confidence levels of the CDR3 loops in the models.

### Web server implementation

The TCRmodel2 web server interface was developed using Python3 and the Flask framework (https://flask.palletsprojects.com/). Users can choose to model a TCR–pMHC complex (Class I or Class II) or an unbound TCR. In both run modes, the users can either provide the target sequences or build the sequences on the fly by selecting the target TCR or MHC genes. The latter option makes possible the modeling of sequences by the information collected from databases such as VDJdb (31) without the need to manually build TCR and MHC sequences. As an additional option, users can input TCR, peptide and MHC sequences in a FASTA format file. Input TCR sequences are preprocessed using the ANARCI tool (2) to identify and keep only their variable domains. In the case of MHC Class II modeling, peptide sequences are truncated to 11-mers based on 9-mer core sequences identified by the NetMHCIIPan program (5). Reference TCR and MHC protein sequences were obtained from the IMGT database (3). Modeling jobs are submitted to a computing cluster with queue management and processed on a dedicated GPU node. Output models are postprocessed by renumbering TCR sequences following the Aho numbering scheme (32) using ANARCI, renaming chains according to TCR3d scheme and aligning models to the top-ranked model by the pMHC chains as reference.

### Benchmarking

#### Benchmark assembly

Experimentally determined TCR–pMHC complex structures used for benchmarking were obtained from the TCR3d database (33). TCR–pMHC complex structures were selected as benchmark cases based on the following criteria: (i) release date after the selected cutoff date (30 April 2018 or 30 September 2021); (ii) structure resolution of 3.25 Å or better; (iii) no redundancy with any TCR–pMHC complex structure from on or before the respective cutoff date (30 April 2018 or 30 September 2021); and (iv) no redundancy with other structures within the benchmark. Redundancy between a pair of complexes was defined as TCR Vα or Vβ sequence identity of ≥95%, or V domain sequence identity of ≥92%, with a complex of the same class (Class I or Class II). Additionally, complexes containing peptides with modified amino acids (e.g. citrullination, lipopeptide) were excluded from the benchmark sets.

#### Accuracy assessment

TCR–pMHC models were assessed using Critical Assessment of Predicted Interactions (CAPRI) criteria (34), which are based on a combination of fraction of native interface residue contacts present in the model (Fnat), interface backbone root-mean-square distance (RMSD) between model and native structure (I-RMSD), and ligand RMSD between model and native structure (L-RMSD). Those metrics were calculated by the DockQ program (35). Additionally, we separately assessed the peptide–MHC interface in models using DockQ and CAPRI peptide docking criteria (36) to identify models with partially or fully displaced peptides (corresponding to CAPRI peptide Incorrect peptide–MHC accuracy). Assessment of unbound TCR models was performed through calculation of backbone RMSD between model and native CDR loop residues after superposition of framework residues using the ProFit program (v3.1). CDR loop residue ranges were based on TCR3d loop definitions.

#### Correlations and ROC AUC calculations

Pearson correlations and their *P*-values were calculated with ggpubr package in R (r-project.org), and receiver operating characteristic (ROC) area under the curve (AUC) calculations were performed using the pROC package (37) in R.

### Other modeling servers and tools

All other modeling tools used for comparison were run with default parameters, using sequences or gene names as input, as required for the respective programs. TCRFlexDock was run using the published Rosetta-based pipeline (21), generating 1000 models per complex, with modeled pMHC and unbound TCR generated by TCRmodel2 (with 30 April 2018 template date cutoff) as input. ImmuneScape (22) and TCRpMHCmodels (23) were run from the respective web server interfaces. TCRDock (16) was downloaded from GitHub and run locally; based on its pipeline, three TCRDock models were generated per complex, and models were ranked by their predicted aligned error (PAE) scores to select the top model. AlphaFold v2.2 and v2.3 were run locally with default parameters and databases for multimer protein prediction, except with five models generated per complex in order to compare with TCRmodel2, and a template date cutoff corresponding to the benchmark set being tested (30 April 2018 or 30 September 2021).

## RESULTS

### TCRmodel2 interface

#### Overview

TCRmodel2 allows users to submit TCR, peptide and MHC sequences to model TCR–pMHC complex structures through its main server interface, and it is able to model Class I and Class II complex structures. As with the original TCRmodel interface (18), users can enter all sequences directly, or generate TCR and MHC sequences from sets of human and mouse genes. As noted in the ‘Materials and Methods’ section, the TCRmodel2 algorithm is based on an adaptation of AlphaFold2, with focused databases of TCR and MHC sequences to speed up MSA feature building, optimization of the TCR template selection and utilization of peptide–MHC complex structures as templates to improve AlphaFold’s peptide–MHC modeling accuracy. Users have the option of performing Amber relaxation of models in AlphaFold, which, as noted in the AlphaFold publication, can improve local geometries in some models (e.g. remove side chain clashes) but will not markedly affect overall model accuracies (24). Currently, TCRmodel2 supports models of TCR complexes with peptide–MHC, and not TCR complexes with MHC-like molecules CD1 and MR1, due to the small molecule and lipid antigens presented by those molecules (38) that are not supported in AlphaFold.

#### Timing

The TCRmodel2 server TCR–pMHC modeling jobs take ∼15 min on average, using a dedicated NVIDIA Titan RTX GPU and generating five ranked TCR–pMHC models. Modeling of unbound TCRs requires ∼12 min to generate five models. Use of model relaxation for the models, which can remove clashes but does not impact overall model accuracy, takes ∼1–2 min (included in the above times). In contrast, generation of five TCR–pMHC models using the standard AlphaFold pipeline on the same computer cluster takes ∼5–7 h, of which over 90% of the time is spent in the feature generation and MSA building stage.

### TCRmodel2 modeling accuracy

#### Initial benchmarking

**Figure 1.**
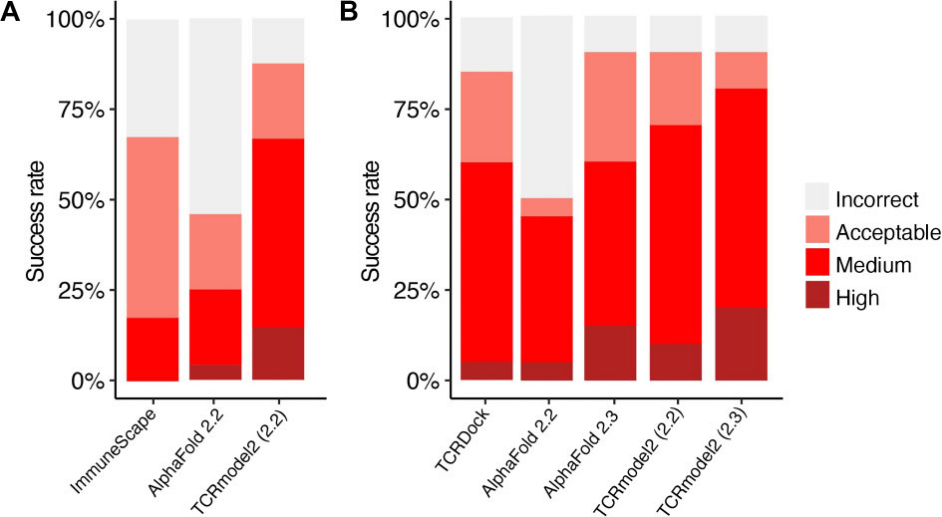
Success rate of TCRmodel2 and comparison with other modeling algorithms. (**A**) Modeling success comparison of AlphaFold 2.2, TCRmodel2 with the AlphaFold 2.2 model (2.2) and ImmuneScape on the initial set of 48 TCR–pMHC benchmarking cases. A template date cutoff of 30 April 2018 was applied. Due to technical issues with 8 cases (job failures, template/case overlap, or MHC allele not available), ImmuneScape success rate calculations are for a subset of 40 cases. (**B**) Modeling success comparison of AlphaFold 2.2, AlphaFold 2.3, TCRmodel2 with the AlphaFold 2.2 model (2.2), TCRmodel2 with the AlphaFold 2.3 model (2.3) and TCRDock on a recently released set of 20 TCR–pMHC structures. Modeling success denotes the success rate of top-ranked prediction if multiple predictions were produced by the modeling algorithm. Three predictions were generated per case by TCRDock, and were ranked by AlphaFold pAE score. For AlphaFold and TCRmodel runs, one prediction per deep learning model was generated, resulting in five predictions per case, ranked by model confidence score. All models were assessed by CAPRI criteria of Incorrect, Acceptable, Medium and High accuracy.

To benchmark the TCR–pMHC modeling accuracy of TCRmodel2, we assembled a set of nonredundant TCR–pMHC structures from TCR3d that were released after 30 April 2018, with the date cutoff selected to avoid overlap with the AlphaFold (v2.2) model training set. Nonredundancy and other criteria for benchmark case selection are detailed in the ‘Materials and Methods’ section. In total, we identified 48 test cases, including 32 Class I complexes and 16 Class II complexes (Supplementary Table S1). Comparison of modeling accuracy of TCRmodel2 with the AlphaFold 2.2 model against AlphaFold 2.2 (Figure 1A and Supplementary Table S1) shows that TCRmodel2 has higher accuracy, achieving a Medium or High CAPRI accuracy model for over 50% of cases. For several cases, such as 6R0E, 6R2L, 6ULN and 7L1D, TCRmodel2 outperformed AlphaFold 2.2, where the latter method improperly modeled the peptide in the interface (Supplementary Table S1), indicating that the pMHC structure templates used by TCRmodel2 likely enabled improved accuracy. Both AlphaFold-based methods outperformed the previously developed template-based TCR–pMHC modeling methods ImmuneScape (22) (Figure 1A and Supplementary Table S1) and TCRpMHCmodels (23) (which only generates Class I TCR–pMHC models; Supplementary Table S1 and Supplementary Figure S1), as well as the TCR–pMHC docking algorithm, TCRFlexDock (21) (Supplementary Table S1). Details regarding the CDR loop accuracies of the TCR–pMHC models and individual accuracy metrics are provided in Supplementary Tables S2 and S3, respectively.

#### Benchmarking updated model

After the recent release of a new AlphaFold model and algorithm (v2.3), which includes an updated training set of structures (up to 30 September 2021) and additional recycling iterations during modeling (https://github.com/deepmind/alphafold/blob/main/docs/technical_note_v2.3.0.md), we implemented TCRmodel2 with the AlphaFold 2.3 model and pipeline to test whether it would lead to accuracy improvement over TCRmodel2 with the AlphaFold 2.2 model. This was benchmarked using 20 TCR–pMHC test cases with release dates after September 2021 (to ensure no overlap with the AlphaFold 2.3 training set), which is a subset of the original benchmark set (14 Class I complexes and 6 Class II complexes; Supplementary Table S4). Modeling performance of TCRmodel 2.2, TCRmodel 2.3, AlphaFold 2.2 and AlphaFold 2.3 was assessed on this recently released benchmark set, along with TCRDock, which is an AlphaFold-based algorithm to model TCR–pMHC complexes that uses a fine-tuned TCR–pMHC model and TCR–pMHC complex templates (16) (Figure 1B and Supplementary Table S4). For this set, the AlphaFold and TCRmodel2 methods were permitted to use TCR and MHC structural templates from on or before the September 2021 date cutoff, versus the April 2018 template date cutoff used for the larger benchmark set. Based on this comparison, the AlphaFold 2.3 model and pipeline led to improved performance, with AlphaFold 2.3 outperforming AlphaFold 2.2, and TCRmodel2 with the AlphaFold 2.3 model outperformed the previous TCRmodel2 implementation (with the AlphaFold 2.2 model). TCRmodel2 (AlphaFold 2.3 model) achieved 20% success for High accuracy near-native models, and showed superior modeling accuracy on the benchmark versus AlphaFold 2.3 and TCRDock. One case for which TCRmodel2 and AlphaFold 2.3 outperformed TCRDock is 7RRG (Supplementary Table S4); as that complex has an unusual TCR docking orientation [74° TCR–pMHC crossing angle, according to TCR3d (33)], it may be more amenable to approaches such as TCRmodel2 and AlphaFold that do not utilize TCR–pMHC structural templates for TCR–pMHC orientation, versus TCRDock that uses TCR–pMHC orientations from experimentally determined complex structures as templates. Given its superior modeling performance, TCRmodel2 with the AlphaFold 2.3 model was selected for use in the TCRmodel2 server.

#### Unbound TCRs

We also benchmarked the use of TCRmodel2 to model individual TCR structures (without pMHC), in comparison with TCRmodel (which uses structural templates to generate models) and AlphaFold (Supplementary Table S6 and Supplementary Figure S2). We found that TCRmodel2 showed commensurate accuracy with AlphaFold (v2.2 and v2.3), while both AlphaFold and TCRmodel2 showed superior performance to TCRmodel, particularly for CDR3 loops that are more challenging to model [as observed during the initial TCRmodel benchmarking (18)] yet critical for peptide recognition. This demonstrates that deep learning-based approaches can overcome CDR3 loop modeling challenges faced by approaches that are fully or mostly reliant on structural templates, including CDR3 loop structural diversity, limited structural templates available, and nontrivial relationships between loop sequences and structures (for accurate template identification).

### Model confidence scoring

**Figure 2.**
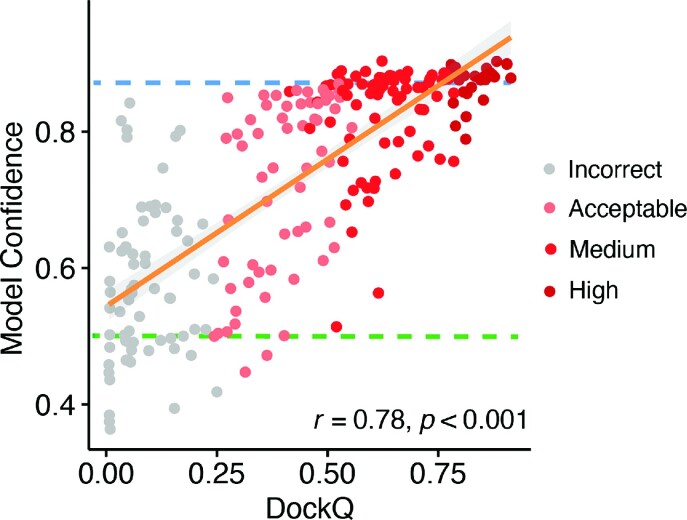
Comparison of model confidence score and model accuracy. Model confidence scores of all five models for 48 cases generated by TCRmodel2 are shown in comparison with model accuracy with respect to the experimentally determined structure [DockQ score (35)], with each model represented as a point and colored by CAPRI accuracy level. Pearson’s correlation coefficient and the associated *P*-value are noted on the lower right corner, and the orange line represents the linear fit (with 95% confidence area in gray). The dashed blue line indicates a suggested ranking confidence cutoff (model confidence = 0.85) for identification of near-native predictions, based on maximization of specificity for discriminating Incorrect and Acceptable versus Medium and High CAPRI accuracy models. The dashed green line indicates the lower bound of the ranking confidence score for models with Acceptable or higher accuracy (model confidence = 0.49).

Given that AlphaFold outputs model confidence estimates that are generally correlated with model accuracy (24,25), we tested the use of AlphaFold confidence estimates in discriminating accurate versus inaccurate TCR–pMHC models in TCRmodel2 (Supplementary Table S6). To maximize the amount of data for this comparison, the models from TCRmodel2 for the larger set of 48 cases (using TCRmodel2 with AlphaFold 2.2 model), and five models per case, were considered. Based on the ROC AUC values reported in Supplementary Table S5, the overall model confidence score, which is a combination of ipTM and pTM scores, was found to provide very good discrimination of Medium and High models versus Incorrect (AUC = 0.97). All of the other confidence metrics tested showed similar AUC values; thus, we focused on the model confidence score for further analysis. When comparing the model confidence score versus model accuracy (Figure 2), a relatively high correlation was observed between model confidence and the model accuracy (denoted by DockQ score) (*r* = 0.75; *P* < 0.001). Model confidence scores also showed significant correlations with individual accuracy metrics Fnat, L-RMSD and I-RMSD (Supplementary Figure S3). Based on analysis of model accuracy discrimination using this score and our benchmark, we have determined model confidence score cutoffs of 0.85 and 0.49 for denoting likely accurate models (≥0.85) or likely inaccurate models (≤0.49) (shown as dashed lines in Figure 2); these cutoffs can be referred to by TCRmodel2 users to gauge the presence of a likely accurate model in the set of five produced by TCRmodel2. As also used by AlphaFold, the model confidence score is used by TCRmodel2 to rank the five models for each TCR–pMHC complex.

To further assess expected model confidence for structurally uncharacterized TCR–pMHC complexes, we used TCRmodel2 to model additional Class I and Class II complexes obtained from the VDJdb database (31). The distributions of model confidence scores for Class I (*N* = 414) and Class II (*N* = 47) complexes are shown in Supplementary Figure S4, indicating that many complexes have top-ranked models in the high confidence range (≥0.85 model confidence score), with 30% of the Class I complexes and 47% of the Class II complexes at that level. With a slightly more permissive threshold (model confidence ≥0.75), TCRmodel2 generated top-ranked models for 77% and 89% of the Class I and Class II complexes, respectively.

### TCR complex modeling examples

**Figure 3.**
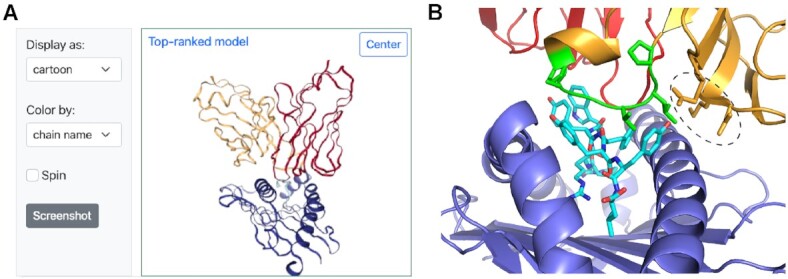
Example TCR–pMHC modeling output from TCRmodel2. (**A**) A TCR–pMHC complex with a human TCR, SARS-CoV-2 nucleocapsid epitope and HLA-B*07:02 MHC from a recent study (39) was modeled using TCRmodel2. The visualization of the top-ranked model from the Results page is shown, with TCR α chain red, β chain orange, peptide cyan and MHC blue. (**B**) The interface between the TCR and pMHC of the top-ranked model is shown, with TCR, peptide and MHC chains colored as in panel (A), and shared CDR3β motif residues (sequence: PLVGAP) colored green and shown as sticks. Peptide residues are shown as sticks, and TCR CDR1β and CDR2β residues interacting with the MHC and/or peptide are shown as sticks and circled. Structure visualized using PyMOL (Schrödinger, Inc.).

As an example of TCR–pMHC complex modeling in TCRmodel2, the server was used to predict the structure of a human TCR in complex with an immunodominant SARS-CoV-2 nucleocapsid epitope presented by the Class I HLA-B*07:02 MHC. The complex has not been structurally characterized, nor have any complexes with TCRs targeting that epitope, and its sequence is from a set of TCRs from COVID-19 recovered and unexposed donors reported in a recent study to bind that peptide (sequence: SPRWYFYYL) and MHC (39). Of note, the TCR contains the TRBV27 germline gene and a long CDR3β sequence (18 residues) containing a sequence motif (PxxGxP); those features were found by the authors to be associated with TCRs targeting that epitope. After input of the germline gene and TCR CDR3 sequences reported by the authors (α: TRAV35/TRAJ39, CAGQLNAGNMLTF; β: TRBV27/TRBJ2-4, CASAPLVGAPEAKNIQYF), along with the epitope sequence and MHC, TCRmodel2 was used to generate five structural models of the complex. The server identified the unbound pMHC structure of the target peptide and MHC (PDB code 7LG0) among its four pMHC templates, and the TCR α and β chain templates with highest sequence identities to the target sequences (each with 89% identity) contain germline genes matching the target (α: 5W1V, TRAV35; β: 6VQO, TRBV27). The top-ranked model (Figure 3) had a high model confidence score (0.86), and inspection of the predicted interface with pMHC (Figure 3B) showed extensive interaction of the CDR3β, and the PxxGxP motif residues (PLVGAP) in particular, with the peptide, as well as the TRBV27-encoded germline loops making extensive interactions with the MHC. This provides a possible mechanistic explanation for the observed preference for TRBV27 in TCRs targeting that epitope, as well as the observed CDR3β sequence motif within the long CDR3β loop.

For a second example, TCRmodel2 was used to model the structure of a Class II TCR–pMHC interaction with a tumor-infiltrating lymphocyte TCR (named 4285-TCR1) that was found to target the common Class II MHC allele HLA-DRB1*13:01 and a p53 neoantigen with the R175H mutation (40). While structures of Class I TCR–pMHC complexes with the p53 neoantigen mutation have been reported (41,42), no Class II structures with that mutation have yet been described. To elucidate that mode of CD4^+^ T cell recognition of the p53 R175H hotspot mutation, we input the TCR Vα and Vβ sequences into the TCRmodel2 submission page, along with a p53 peptide sequence containing the mutant residue (TEVVR**H**CPHHERCSD; mutant histidine in bold), and selected the HLA-DRA*01:01 and HLA-DRB1*13:01 MHC genes. The Results page from TCRmodel2 included the top-ranked model of that TCR–pMHC complex (Supplementary Figure S5A), which has a high confidence score (0.88). Downloading the PDB structure of the top-ranked model and visualization of its structure indicates that the mutant histidine residue is located directly at the interface with the TCR, engaging both α and β chains, suggesting a possible mechanism for the neoantigen specificity of that TCR (Supplementary Figure S5B).

## DISCUSSION

The TCRmodel2 web server provides the community with a deep learning method to accurately model structures of TCRs and TCR–pMHC complexes. Its TCR–pMHC accuracy is higher than AlphaFold, it runs faster and without the need for dedicated computing resources, and it provides a submission and output interface designed for TCR and TCR–pMHC modeling. TCRmodel2 is distinguished from another recently reported AlphaFold-based TCR–pMHC modeling method (16), as it does not rely on fine-tuning of the AlphaFold model or TCR–pMHC complex templates, in addition to its availability as a web server versus a command-line program.

Future possible developments of TCRmodel2 can address improving the accuracy of confidence estimates, and increasing the overall success rate, including the generation of near-native (CAPRI High criteria) accuracy models, through additional optimizations of the modeling pipeline. Additional testing and developments may focus on application of TCRmodel2 for related complexes of interest, such as TCR-mimic antibodies (43), which engage pMHC targets and are becoming increasingly of interest as therapeutics. Modeling of such complexes would likely entail limited, if any, adaptations to the current TCRmodel2 framework, including a possible expansion of the MSA database to optimize antibody sequence hits. Given the recent utilization deep learning structure prediction methods to design new proteins and interactions (44,45), it may be possible that TCRmodel2 or similar methods can be used in future studies to design and optimize TCRs to target antigens of interest.

## DATA AVAILABILITY

The TCRmodel2 code is available on GitHub (https://github.com/piercelab/tcrmodel2) and Zenodo (https://doi.org/10.5281/zenodo.7853278). Structural models from the benchmarking reported in this manuscript are available from the authors upon request.

## Supplementary Material

gkad356_Supplemental_File

## Notes

Present address: Ragul Gowthaman, Cyrus Biotechnology, Seattle, WA 98121, USA.
