# Supplementary material for: TCRmodel2: high-resolution modeling of T cell receptor recognition using deep learning
Source: Nucleic Acids Res. 2023 May 4;51(W1):W569–76. doi: 10.1093/nar/gkad356 (PMC10320165; doi:10.1093/nar/gkad356)
Supplement: gkad356_Supplemental_File [file gkad356_supplemental_file.pdf]

Table S1. Modeling accuracy of AlphaFold, TCRmodel2, TCRpMHCmodels, ImmuneScape and TCRFlexDock on the benchmarking set of 48 TCR-pMHC complexes.

PDB	MHC class	Vα %[a]	Vβ %[a]	AlphaFold 2.2[b]		TCRmodel2 (v2.2)[b]		TCRpMHC models[c]	Immune Scape[d]	TCRFlex Dock[e]
				T1	T5	T1	T5			
6l9l	1	91	93	1	1	1	1	1	1	2
6mtm	1	86	92	2	2	2	2	1	2	2
6r2l	1	**87**	**82**	-1	-1	2	2	0	0	0
6rpa	1	**65**	**57**	-1	1	1	1	-	2	1
6rpb	1	87	91	0	0	0	1	2	0	1
6rsy	1	**84**	**89**	2	2	2	3	1	1	1
6tro	1	51	92	0	2	3	3	0	1	1
6uk4	1	**88**	**89**	-1	-1	1	1	1	2	1
6uln	1	88	90	-1	-1	2	2	1	1	2
6vrm	1	89	95	-1	-1	1	2	0	1	0
6vrn	1	86	91	-1	-1	0	0	0	0	0
7n1e	1	52	95	0	0	0	0	-	1	1
5nht	1	95	89	1	2	3	3	2	2	-
6rp9	1	88	90	1	1	2	2	0	0	0
6vmx	1	92	91	-1	2	3	3	1	1	0
6vm8	1	73	95	1	1	1	1	1	1	-
7n6e	1	91	89	1	1	1	1	1	1	1
6zkw	1	89	92	1	2	1	1	0	0	-
7dzm	1	**88**	**88**	2	2	0	0	0	1	-
7ndq	1	89	91	2	2	2	2	0	-	-
7l1d	1	**89**	**89**	-1	-1	2	2	-	1	-
7rrg	1	88	92	0	0	2	2	0	0	-
7na5	1	**62**	**54**	0	1	0	1	0	0	-
7ow5	1	83	90	-1	-1	2	2	1	1	-
7qpj	1	**88**	**88**	3	3	3	3	2	2	-
7phr	1	87	94	3	3	3	3	1	1	-
7nme	1	90	94	2	2	2	2	1	1	-
8d5q	1	66	93	2	2	2	2	1	0	-
8gvb	1	90	90	2	2	2	2	1	0	-
7rk7	1	**88**	**85**	-1	-1	1	1	-	0	-
7n2n	1	91	93	0	0	0	1	-	0	-
8gom	1	91	88	-1	0	1	1	0	1	-
6cql	2	**52**	**55**	-1	2	1	1	-	-	-
6dfx	2	89	93	1	1	2	2	-	0	2
6py2	2	92	58	1	1	2	2	-	0	1
6r0e	2	92	61	-1	-1	3	3	-	2	2
6u3n	2	92	76	-1	2	2	2	-	2	2
6xc9	2	45	93	2	2	2	2	-	1	1
6xco	2	89	94	2	2	3	3	-	1	1
6dfs	2	**67**	**38**	1	1	2	2	-	-	2

6dfw	2	66	93	-1	1	2	2	-	-	1
6px6	2	51	95	0	2	2	2	-	1	-
7rdv	2	64	92	-1	-1	2	2	-	-	-
7sg0	2	93	90	-1	2	2	2	-	-	-
7sg1	2	91	57	-1	-1	2	2	-	-	-
7z50	2	**67**	**59**	-1	-1	2	3	-	-	-
7t2c	2	92	92	2	2	2	2	-	1	-
7t2b	2	91	94	1	2	2	2	-	1	-

All predictions were assessed by CAPRI criteria for Incorrect (denoted by "0"), Acceptable (denoted by "1"), Medium (denoted by "2"), and High (denoted by "3") modeling accuracy. Peptide displacement from the MHC was assessed using CAPRI criteria, and denoted with "-1". Cases without predictions generated are denoted with "-".

[a]Maximum α chain variable domain (Vα) and β chain variable domain (Vβ) sequence identity with any TCR from known TCR-pMHC structures of the same class from on or before the 2018-04-30 date cutoff. Values in bold denote complexes with both chains below a 90% sequence identity level, indicative of structures with limited germline and/or CDR3 sequence match to previously described known structures for both chains.

[b]Five models were generated for each complex, and predictions were ranked by model confidence score. A template date cutoff of 2018-04-30 was applied to disable the use of recently released structures (overlapping with this benchmark set) as templates.

[c]TCRpMHCmodels predicts TCR-pMHC complexes with MHC Class I molecules. Some Class I cases resulted in job failures and are noted with "-".

[d]ImmuneScape job failures, or cases for which the appropriate MHC allele was not found, are noted as "-". Additionally, complex 6cql was excluded from ImmuneScape benchmarking, due to its overlap with the template used by that server.

[e]TCRFlexDock was run on a subset of cases from an early version of the benchmark. Modeled TCR and pMHC structures were used as input.

Table S2. CDR loop modeling accuracy of TCRmodel2 (2.2 AlphaFold model) and TCRmodel2 (2.3 AlphaFold model) for TCR-pMHC structures.

PDB	MHC class	TCRmodel2 (2.2)						TCRmodel2 (2.3)					
		**1α**	**2α**	**3α**	**1β**	**2β**	**3β**	**1α**	**2α**	**3α**	**1β**	**2β**	**3β**
6zkw	1	0.57	0.44	0.56	0.41	0.37	0.98	0.53	0.44	0.35	0.39	0.36	1.09
7na5	1	0.35	0.39	0.78	0.59	1.16	1.07	0.36	0.37	0.77	0.57	1.18	0.93
7ow5	1	2.16	2.16	3.82	0.60	0.48	6.67	2.23	0.74	4.37	0.66	0.45	5.86
7qpj	1	0.40	0.61	2.85	0.43	0.50	1.38	0.49	0.60	2.47	0.31	0.35	1.39
8gvb	1	0.77	0.40	0.77	0.61	0.81	1.51	0.74	0.46	0.69	0.59	1.03	1.37
7phr	1	0.77	0.71	1.44	0.76	1.38	2.04	0.76	0.61	1.65	0.42	1.31	2.00
7ndq	1	0.56	0.64	1.12	0.36	0.36	1.28	0.54	0.64	1.01	0.32	0.30	1.36
7rrg	1	2.16	0.74	3.82	0.37	0.66	0.47	0.50	0.43	1.07	0.36	0.65	0.77
7l1d	1	0.43	0.51	0.52	0.37	0.31	1.00	0.39	0.48	0.54	0.32	0.27	0.87
7dzm	1	0.59	0.85	5.03	0.84	0.42	4.51	0.59	0.88	3.82	0.87	0.43	3.87
7sg1	2	0.52	0.45	2.36	0.26	0.56	0.45	0.45	0.38	4.86	0.32	0.53	1.79
7rdv	2	1.11	0.30	2.45	0.33	0.56	1.21	1.15	0.35	2.79	0.29	0.51	1.05
7z50	2	0.57	0.59	2.08	0.55	0.89	2.31	0.40	0.50	1.91	0.52	0.90	3.18

Modeling accuracy is shown as backbone root-mean-square distance (RMSD) in Ångstroms of the respective CDR loop between model and native structures, after superposition of framework regions.

Table S3. TCR-pMHC modeling accuracy metrics and scores of TCRmodel2 (2.2 AlphaFold model) on the benchmarking set of 48 TCR-pMHC complexes.

PDB	TCR-pMHC complex accuracy						Model confi-dence	pLDDT	pTM	ipTM	TCR-pMHC ipTM[a]	Peptide-MHC accuracy[b]
	DockQ	Fnat	I-RMSD	L-RMSD	Fnonnat	CAPRI						
6l9l/ranked_0	0.50	0.60	2.38	6.48	0.43	Acceptable	0.85	94.27	0.88	0.85	0.82	High
6l9l/ranked_1	0.06	0.00	11.02	19.95	1.00	Incorrect	0.57	87.99	0.66	0.55	0.37	High
6l9l/ranked_2	0.14	0.02	5.83	11.59	0.98	Incorrect	0.56	89.45	0.65	0.53	0.33	High
6l9l/ranked_3	0.05	0.00	10.18	21.55	1.00	Incorrect	0.51	88.28	0.61	0.48	0.27	High
6l9l/ranked_4	0.06	0.00	10.58	19.23	1.00	Incorrect	0.49	88.81	0.60	0.46	0.24	High
6mtm/ranked_0	0.68	0.71	1.64	3.16	0.41	Medium	0.89	93.63	0.91	0.88	0.88	Medium
6mtm/ranked_1	0.66	0.67	1.65	3.33	0.44	Medium	0.88	92.85	0.90	0.88	0.87	Acceptable
6mtm/ranked_2	0.68	0.64	1.57	2.71	0.49	Medium	0.88	92.97	0.90	0.87	0.86	Medium
6mtm/ranked_3	0.76	0.67	1.14	1.48	0.34	Medium	0.88	93.33	0.90	0.87	0.86	Medium
6mtm/ranked_4	0.65	0.59	1.64	2.80	0.49	Medium	0.86	92.55	0.88	0.85	0.83	Medium
6r2l/ranked_0	0.75	0.67	1.07	2.37	0.22	Medium	0.76	91.27	0.80	0.75	0.67	High
6r2l/ranked_1	0.06	0.08	11.39	27.84	0.88	Incorrect	0.62	90.09	0.70	0.60	0.44	High
6r2l/ranked_2	0.25	0.20	3.43	10.40	0.64	Acceptable	0.50	88.89	0.60	0.48	0.24	High
6r2l/ranked_3	0.06	0.07	10.94	27.71	0.80	Incorrect	0.48	88.31	0.59	0.45	0.21	High
6r2l/ranked_4	0.01	0.00	25.32	69.32	1.00	Incorrect	0.46	86.32	0.57	0.43	0.16	High
6rpa/ranked_0	0.41	0.40	3.33	6.16	0.61	Acceptable	0.84	92.82	0.86	0.83	0.80	High
6rpa/ranked_1	0.04	0.04	14.55	27.78	0.94	Incorrect	0.63	89.99	0.71	0.61	0.46	High
6rpa/ranked_2	0.04	0.02	14.38	27.54	0.96	Incorrect	0.63	89.62	0.70	0.61	0.45	High
6rpa/ranked_3	0.09	0.11	10.52	21.17	0.86	Incorrect	0.62	90.37	0.70	0.60	0.46	High
6rpa/ranked_4	0.05	0.05	13.10	25.46	0.93	Incorrect	0.56	88.82	0.65	0.54	0.36	High
6rpb/ranked_0	0.22	0.29	4.98	13.77	0.59	Incorrect	0.66	92.83	0.73	0.65	0.50	High
6rpb/ranked_1	0.24	0.32	4.76	12.64	0.54	Incorrect	0.58	92.21	0.67	0.56	0.37	High
6rpb/ranked_2	0.28	0.24	3.55	9.25	0.53	Acceptable	0.57	91.91	0.66	0.55	0.34	Medium
6rpb/ranked_3	0.20	0.15	4.59	11.44	0.55	Incorrect	0.51	91.07	0.61	0.48	0.24	Medium

6rpb/ranked_4	0.23	0.21	4.24	11.42	0.36	Incorrect	0.51	91.02	0.61	0.48	0.25	Medium
6rsy/ranked_0	0.78	0.77	1.02	2.97	0.21	Medium	0.89	94.42	0.91	0.89	0.88	High
6rsy/ranked_1	0.77	0.74	1.04	3.05	0.17	Medium	0.88	94.02	0.90	0.88	0.86	High
6rsy/ranked_2	0.73	0.69	1.13	3.37	0.26	Medium	0.87	93.68	0.89	0.87	0.84	High
6rsy/ranked_3	0.81	0.78	0.97	2.24	0.12	High	0.86	93.37	0.88	0.85	0.83	High
6rsy/ranked_4	0.01	0.00	23.49	55.40	1.00	Incorrect	0.56	89.19	0.66	0.54	0.36	High
6tro/ranked_0	0.78	0.78	0.99	3.40	0.21	High	0.89	96.24	0.91	0.89	0.87	High
6tro/ranked_1	0.81	0.82	0.93	3.15	0.14	High	0.88	95.90	0.90	0.88	0.86	High
6tro/ranked_2	0.82	0.82	0.94	2.78	0.14	High	0.86	95.21	0.88	0.85	0.82	High
6tro/ranked_3	0.79	0.78	0.97	3.09	0.22	High	0.81	94.10	0.84	0.80	0.73	Medium
6tro/ranked_4	0.79	0.81	1.07	3.12	0.21	Medium	0.76	92.91	0.80	0.75	0.66	High
6uk4/ranked_0	0.46	0.55	2.58	7.44	0.51	Acceptable	0.86	94.37	0.89	0.85	0.85	High
6uk4/ranked_1	0.44	0.52	2.63	7.56	0.48	Acceptable	0.84	93.55	0.87	0.83	0.83	High
6uk4/ranked_2	0.44	0.55	2.65	7.80	0.47	Acceptable	0.81	93.07	0.86	0.80	0.80	High
6uk4/ranked_3	0.14	0.08	6.77	13.64	0.92	Incorrect	0.69	91.29	0.75	0.67	0.56	High
6uk4/ranked_4	0.20	0.14	5.39	10.73	0.86	Incorrect	0.59	90.00	0.67	0.57	0.44	High
6uln/ranked_0	0.54	0.49	2.01	4.61	0.68	Medium	0.85	92.94	0.88	0.85	0.82	Medium
6uln/ranked_1	0.50	0.45	2.31	5.04	0.70	Acceptable	0.85	93.32	0.87	0.84	0.81	High
6uln/ranked_2	0.46	0.47	2.66	5.93	0.71	Acceptable	0.84	92.65	0.87	0.84	0.80	Medium
6uln/ranked_3	0.52	0.49	2.18	5.05	0.67	Acceptable	0.83	92.76	0.86	0.83	0.79	High
6uln/ranked_4	0.06	0.10	14.75	29.29	0.87	Incorrect	0.57	87.71	0.66	0.55	0.36	High
6vrm/ranked_0	0.45	0.50	2.56	6.98	0.43	Acceptable	0.66	92.34	0.73	0.64	0.50	Medium
6vrm/ranked_1	0.52	0.52	2.13	5.65	0.48	Acceptable	0.63	89.91	0.71	0.61	0.47	High
6vrm/ranked_2	0.49	0.44	2.35	5.10	0.47	Acceptable	0.61	90.07	0.69	0.59	0.42	High
6vrm/ranked_3	0.42	0.43	2.37	7.55	0.41	Acceptable	0.58	91.05	0.67	0.56	0.40	Medium
6vrm/ranked_4	0.62	0.52	1.67	3.12	0.22	Medium	0.56	90.00	0.65	0.54	0.34	High
6vrn/ranked_0	0.17	0.19	6.02	14.59	0.83	Incorrect	0.80	92.87	0.84	0.79	0.74	High
6vrn/ranked_1	0.16	0.15	6.06	14.29	0.88	Incorrect	0.79	92.61	0.83	0.78	0.71	High
6vrn/ranked_2	0.16	0.13	5.79	13.33	0.88	Incorrect	0.67	91.41	0.74	0.65	0.50	High

6vrn/ranked_3	0.16	0.13	5.69	13.65	0.87	Incorrect	0.65	91.12	0.72	0.64	0.48	High
6vrn/ranked_4	0.15	0.11	6.02	14.17	0.89	Incorrect	0.56	89.75	0.65	0.53	0.33	High
7n1e/ranked_0	0.11	0.03	7.53	14.31	0.97	Incorrect	0.68	90.52	0.74	0.67	0.54	High
7n1e/ranked_1	0.01	0.00	22.98	59.15	1.00	Incorrect	0.54	88.96	0.64	0.52	0.32	High
7n1e/ranked_2	0.01	0.00	22.86	59.03	1.00	Incorrect	0.50	88.01	0.61	0.48	0.30	High
7n1e/ranked_3	0.01	0.00	22.98	58.78	1.00	Incorrect	0.48	87.91	0.59	0.46	0.24	High
7n1e/ranked_4	0.25	0.05	4.06	7.25	0.73	Incorrect	0.42	88.16	0.53	0.39	0.14	High
5nht/ranked_0	0.78	0.67	0.95	1.78	0.24	High	0.90	94.27	0.91	0.89	0.88	High
5nht/ranked_1	0.79	0.68	0.93	1.67	0.27	High	0.89	94.17	0.91	0.89	0.88	High
5nht/ranked_2	0.79	0.67	0.93	1.53	0.28	High	0.89	94.07	0.91	0.89	0.88	High
5nht/ranked_3	0.73	0.61	1.06	2.33	0.25	Medium	0.88	94.08	0.90	0.88	0.85	High
5nht/ranked_4	0.76	0.64	1.02	1.78	0.28	Medium	0.88	93.61	0.90	0.87	0.85	High
6rp9/ranked_0	0.61	0.54	1.69	3.65	0.54	Medium	0.82	94.02	0.85	0.81	0.75	Medium
6rp9/ranked_1	0.61	0.54	1.69	3.63	0.53	Medium	0.73	93.02	0.78	0.71	0.60	Medium
6rp9/ranked_2	0.09	0.10	9.17	20.98	0.91	Incorrect	0.61	91.02	0.69	0.59	0.41	Medium
6rp9/ranked_3	0.29	0.22	3.75	8.22	0.74	Acceptable	0.52	89.39	0.62	0.49	0.27	Medium
6rp9/ranked_4	0.10	0.07	8.17	17.98	0.90	Incorrect	0.48	88.74	0.59	0.45	0.20	Medium
6vmx/ranked_0	0.83	0.74	0.83	1.45	0.25	High	0.87	94.19	0.89	0.86	0.83	Medium
6vmx/ranked_1	0.82	0.72	0.84	1.57	0.23	High	0.86	93.85	0.88	0.85	0.82	Medium
6vmx/ranked_2	0.81	0.74	0.91	1.46	0.25	High	0.82	93.29	0.85	0.81	0.76	Medium
6vmx/ranked_3	0.81	0.72	0.84	1.88	0.26	High	0.79	92.57	0.83	0.78	0.71	Medium
6vmx/ranked_4	0.73	0.63	1.07	3.01	0.20	Medium	0.78	92.43	0.82	0.77	0.69	Medium
6vm8/ranked_0	0.27	0.25	3.59	9.99	0.64	Acceptable	0.85	93.94	0.88	0.84	0.81	High
6vm8/ranked_1	0.30	0.32	3.50	10.09	0.61	Acceptable	0.80	93.37	0.83	0.79	0.73	High
6vm8/ranked_2	0.27	0.29	3.67	10.73	0.59	Acceptable	0.79	93.39	0.83	0.78	0.72	High
6vm8/ranked_3	0.31	0.35	3.51	10.07	0.55	Acceptable	0.78	92.95	0.82	0.77	0.70	High
6vm8/ranked_4	0.01	0.00	20.01	70.42	0.00	Incorrect	0.37	87.62	0.51	0.34	0.08	High
7n6e/ranked_0	0.53	0.57	2.26	5.63	0.36	Acceptable	0.86	94.29	0.88	0.86	0.82	High
7n6e/ranked_1	0.40	0.47	3.09	7.95	0.41	Acceptable	0.82	93.40	0.85	0.81	0.77	High

7n6e/ranked_2	0.56	0.65	2.35	5.17	0.37	Acceptable	0.81	92.84	0.84	0.80	0.77	High
7n6e/ranked_3	0.50	0.52	2.29	5.60	0.41	Acceptable	0.76	92.53	0.80	0.74	0.68	High
7n6e/ranked_4	0.01	0.00	24.05	58.59	1.00	Incorrect	0.58	91.31	0.67	0.56	0.37	High
6zkw/ranked_0	0.53	0.69	2.65	6.21	0.32	Acceptable	0.85	94.83	0.88	0.84	0.79	Medium
6zkw/ranked_1	0.44	0.48	2.49	7.44	0.53	Acceptable	0.72	91.83	0.78	0.70	0.58	Medium
6zkw/ranked_2	0.51	0.52	2.14	5.97	0.44	Acceptable	0.67	90.73	0.74	0.65	0.50	Medium
6zkw/ranked_3	0.40	0.41	2.81	7.36	0.54	Acceptable	0.65	90.90	0.72	0.63	0.47	Medium
6zkw/ranked_4	0.40	0.28	2.35	6.37	0.45	Acceptable	0.50	88.63	0.61	0.47	0.25	Medium
7dzm/ranked_0	0.04	0.02	8.84	35.06	0.98	Incorrect	0.65	91.27	0.72	0.63	0.49	High
7dzm/ranked_1	0.06	0.00	7.92	20.09	1.00	Incorrect	0.61	88.14	0.69	0.59	0.43	High
7dzm/ranked_2	0.04	0.02	9.05	29.02	0.96	Incorrect	0.50	89.17	0.60	0.47	0.23	High
7dzm/ranked_3	0.01	0.00	23.41	67.91	1.00	Incorrect	0.38	86.52	0.51	0.35	0.08	High
7dzm/ranked_4	0.01	0.00	19.63	58.44	1.00	Incorrect	0.36	85.61	0.51	0.33	0.14	High
7ndq/ranked_0	0.55	0.53	2.23	4.35	0.55	Medium	0.87	92.68	0.89	0.86	0.84	Acceptable
7ndq/ranked_1	0.51	0.45	2.36	4.49	0.59	Medium	0.87	92.29	0.89	0.86	0.84	Acceptable
7ndq/ranked_2	0.50	0.45	2.56	4.50	0.60	Medium	0.87	92.68	0.89	0.86	0.84	Acceptable
7ndq/ranked_3	0.48	0.42	2.39	5.19	0.58	Acceptable	0.82	91.75	0.85	0.81	0.76	Acceptable
7ndq/ranked_4	0.49	0.47	2.36	5.50	0.56	Acceptable	0.77	91.14	0.81	0.76	0.68	Acceptable
7l1d/ranked_0	0.56	0.49	2.52	2.04	0.52	Medium	0.88	91.52	0.89	0.88	0.87	Medium
7l1d/ranked_1	0.41	0.35	3.84	4.79	0.64	Medium	0.86	91.06	0.88	0.85	0.83	High
7l1d/ranked_2	0.48	0.42	2.88	4.29	0.57	Medium	0.84	90.76	0.87	0.84	0.81	Medium
7l1d/ranked_3	0.51	0.43	3.37	2.50	0.58	Medium	0.81	89.86	0.84	0.81	0.76	Medium
7l1d/ranked_4	0.46	0.41	3.00	4.61	0.59	Medium	0.80	90.61	0.84	0.80	0.74	High
7rrg/ranked_0	0.55	0.43	1.87	3.86	0.58	Medium	0.79	91.94	0.83	0.78	0.72	Medium
7rrg/ranked_1	0.58	0.44	1.77	3.37	0.49	Medium	0.73	90.84	0.78	0.71	0.61	Medium
7rrg/ranked_2	0.54	0.41	1.99	3.62	0.57	Medium	0.69	90.61	0.75	0.68	0.55	Medium
7rrg/ranked_3	0.08	0.08	9.79	20.34	0.92	Incorrect	0.67	90.62	0.73	0.65	0.52	Medium
7rrg/ranked_4	0.11	0.11	8.48	17.85	0.86	Incorrect	0.57	88.85	0.66	0.55	0.36	Medium
7na5/ranked_0	0.03	0.02	15.39	30.73	0.98	Incorrect	0.82	92.88	0.85	0.81	0.76	High

7na5/ranked_1	0.05	0.06	15.38	30.78	0.95	Incorrect	0.80	92.45	0.84	0.79	0.73	High
7na5/ranked_2	0.34	0.33	3.43	7.81	0.68	Acceptable	0.80	92.73	0.83	0.79	0.74	High
7na5/ranked_3	0.05	0.06	15.32	30.66	0.95	Incorrect	0.79	92.30	0.83	0.78	0.71	High
7na5/ranked_4	0.05	0.06	17.33	31.80	0.93	Incorrect	0.63	89.32	0.70	0.61	0.47	High
7ow5/ranked_0	0.69	0.64	1.26	3.80	0.35	Medium	0.80	92.84	0.84	0.79	0.71	Acceptable
7ow5/ranked_1	0.10	0.02	8.59	14.69	0.98	Incorrect	0.69	91.37	0.75	0.67	0.54	Medium
7ow5/ranked_2	0.05	0.03	12.29	24.80	0.93	Incorrect	0.56	90.03	0.65	0.53	0.30	Medium
7ow5/ranked_3	0.05	0.03	12.24	24.49	0.92	Incorrect	0.55	90.00	0.65	0.53	0.30	Medium
7ow5/ranked_4	0.05	0.02	12.06	24.43	0.96	Incorrect	0.49	87.36	0.60	0.47	0.21	Medium
7qpj/ranked_0	0.87	0.75	0.60	1.09	0.18	High	0.89	95.31	0.90	0.88	0.86	High
7qpj/ranked_1	0.87	0.78	0.62	1.18	0.17	High	0.88	94.99	0.90	0.88	0.85	Medium
7qpj/ranked_2	0.88	0.77	0.56	0.99	0.19	High	0.88	95.01	0.90	0.88	0.85	High
7qpj/ranked_3	0.91	0.87	0.55	0.84	0.14	High	0.88	95.26	0.90	0.87	0.85	High
7qpj/ranked_4	0.33	0.44	3.86	10.16	0.47	Acceptable	0.82	94.07	0.85	0.81	0.75	Medium
7phr/ranked_0	0.87	0.77	0.64	1.21	0.13	High	0.88	93.71	0.90	0.88	0.87	High
7phr/ranked_1	0.85	0.77	0.72	1.22	0.13	High	0.88	93.85	0.90	0.88	0.87	High
7phr/ranked_2	0.86	0.79	0.75	1.41	0.17	High	0.88	93.81	0.90	0.87	0.87	High
7phr/ranked_3	0.85	0.76	0.73	1.35	0.16	High	0.88	93.78	0.90	0.87	0.87	High
7phr/ranked_4	0.85	0.76	0.70	1.26	0.11	High	0.85	92.99	0.87	0.84	0.83	High
7nme/ranked_0	0.62	0.71	1.88	4.63	0.33	Medium	0.90	94.91	0.92	0.90	0.89	Medium
7nme/ranked_1	0.62	0.64	1.78	4.31	0.35	Medium	0.88	94.28	0.90	0.87	0.85	High
7nme/ranked_2	0.62	0.72	1.89	4.76	0.32	Medium	0.88	94.38	0.90	0.87	0.85	High
7nme/ranked_3	0.64	0.72	1.78	4.47	0.36	Medium	0.88	94.51	0.90	0.87	0.85	High
7nme/ranked_4	0.04	0.05	17.46	32.89	0.88	Incorrect	0.50	89.20	0.60	0.48	0.23	High
8d5q/ranked_0	0.62	0.55	1.73	3.21	0.62	Medium	0.87	92.01	0.89	0.87	0.83	Acceptable
8d5q/ranked_1	0.61	0.51	1.75	3.11	0.62	Medium	0.87	92.00	0.89	0.86	0.83	Acceptable
8d5q/ranked_2	0.63	0.55	1.73	2.87	0.60	Medium	0.87	91.91	0.89	0.86	0.83	Acceptable
8d5q/ranked_3	0.62	0.53	1.75	2.81	0.57	Medium	0.86	91.84	0.89	0.86	0.82	Acceptable
8d5q/ranked_4	0.61	0.51	1.55	3.96	0.57	Medium	0.72	89.52	0.77	0.70	0.58	Medium

8gvb/ranked_0	0.53	0.53	2.33	4.63	0.45	Medium	0.89	94.15	0.91	0.89	0.87	High
8gvb/ranked_1	0.52	0.52	2.31	4.88	0.45	Medium	0.88	93.81	0.90	0.88	0.86	High
8gvb/ranked_2	0.53	0.52	2.25	4.71	0.47	Medium	0.88	93.40	0.90	0.87	0.85	High
8gvb/ranked_3	0.48	0.42	2.39	5.20	0.52	Acceptable	0.84	92.85	0.87	0.83	0.79	High
8gvb/ranked_4	0.01	0.00	25.35	69.60	1.00	Incorrect	0.45	88.34	0.57	0.42	0.14	High
7rk7/ranked_0	0.50	0.62	2.23	7.61	0.41	Acceptable	0.85	91.21	0.87	0.84	0.82	High
7rk7/ranked_1	0.47	0.56	2.29	7.67	0.42	Acceptable	0.82	90.84	0.85	0.81	0.77	High
7rk7/ranked_2	0.38	0.42	2.75	8.90	0.56	Acceptable	0.75	89.55	0.79	0.74	0.66	High
7rk7/ranked_3	0.45	0.55	2.30	8.24	0.46	Acceptable	0.75	90.18	0.79	0.74	0.66	High
7rk7/ranked_4	0.43	0.50	2.39	8.21	0.41	Acceptable	0.65	88.56	0.72	0.64	0.49	High
7n2n/ranked_0	0.05	0.09	16.25	33.23	0.92	Incorrect	0.84	94.68	0.87	0.84	0.79	High
7n2n/ranked_1	0.04	0.04	17.03	32.79	0.96	Incorrect	0.62	90.51	0.70	0.61	0.45	High
7n2n/ranked_2	0.27	0.21	3.71	9.50	0.73	Acceptable	0.61	91.32	0.69	0.59	0.40	High
7n2n/ranked_3	0.06	0.11	16.41	33.42	0.88	Incorrect	0.56	90.35	0.65	0.54	0.33	High
7n2n/ranked_4	0.36	0.33	3.12	7.59	0.56	Acceptable	0.56	90.48	0.65	0.53	0.34	High
8gom/ranked_0	0.53	0.59	2.54	5.26	0.39	Acceptable	0.87	93.35	0.89	0.87	0.85	High
8gom/ranked_1	0.49	0.53	2.49	5.94	0.40	Acceptable	0.86	93.48	0.88	0.86	0.84	High
8gom/ranked_2	0.08	0.06	6.16	22.65	0.91	Incorrect	0.69	91.41	0.75	0.67	0.54	High
8gom/ranked_3	0.24	0.17	3.06	11.17	0.46	Acceptable	0.50	89.64	0.60	0.47	0.24	High
8gom/ranked_4	0.15	0.06	4.03	13.63	0.83	Incorrect	0.39	87.65	0.52	0.36	0.18	High
6cql/ranked_0	0.35	0.28	2.29	9.26	0.59	Acceptable	0.59	89.71	0.68	0.57	0.38	Medium
6cql/ranked_1	0.29	0.20	2.55	9.91	0.68	Acceptable	0.54	87.82	0.63	0.51	0.28	Medium
6cql/ranked_2	0.27	0.20	2.86	10.30	0.69	Acceptable	0.51	86.49	0.61	0.48	0.25	Medium
6cql/ranked_3	0.17	0.05	4.97	10.65	0.88	Incorrect	0.50	87.02	0.60	0.47	0.22	Medium
6cql/ranked_4	0.19	0.10	3.92	11.56	0.71	Incorrect	0.47	87.27	0.58	0.44	0.19	Medium
6dfx/ranked_0	0.59	0.49	1.58	4.13	0.28	Medium	0.70	91.95	0.76	0.68	0.57	High
6dfx/ranked_1	0.15	0.06	4.94	13.20	0.82	Incorrect	0.50	88.30	0.60	0.47	0.22	High
6dfx/ranked_2	0.36	0.22	2.71	6.52	0.38	Acceptable	0.47	88.70	0.58	0.44	0.21	High
6dfx/ranked_3	0.05	0.00	13.38	21.42	1.00	Incorrect	0.47	88.20	0.57	0.44	0.18	High

6dfx/ranked_4	0.06	0.00	9.88	20.83	1.00	Incorrect	0.46	87.67	0.57	0.43	0.17	High
6py2/ranked_0	0.57	0.45	1.93	3.33	0.36	Medium	0.84	92.40	0.87	0.83	0.79	High
6py2/ranked_1	0.35	0.38	3.80	7.89	0.65	Acceptable	0.73	90.76	0.78	0.72	0.61	High
6py2/ranked_2	0.36	0.37	3.63	7.31	0.63	Acceptable	0.70	89.84	0.76	0.68	0.55	High
6py2/ranked_3	0.33	0.35	3.92	8.26	0.64	Acceptable	0.60	88.82	0.68	0.59	0.41	High
6py2/ranked_4	0.32	0.28	3.52	8.04	0.56	Acceptable	0.58	88.23	0.66	0.56	0.35	High
6r0e/ranked_0	0.86	0.85	0.84	1.59	0.35	High	0.89	95.71	0.91	0.89	0.87	High
6r0e/ranked_1	0.74	0.68	1.19	2.68	0.38	Medium	0.88	94.81	0.90	0.88	0.86	High
6r0e/ranked_2	0.83	0.85	0.93	2.39	0.29	High	0.88	95.56	0.90	0.88	0.85	High
6r0e/ranked_3	0.80	0.72	0.93	1.91	0.37	High	0.88	94.92	0.90	0.87	0.85	High
6r0e/ranked_4	0.72	0.68	1.23	3.32	0.33	Medium	0.83	93.45	0.86	0.82	0.78	High
6u3n/ranked_0	0.68	0.63	1.43	3.15	0.35	Medium	0.86	93.34	0.88	0.86	0.82	High
6u3n/ranked_1	0.62	0.56	1.53	4.26	0.41	Medium	0.86	93.34	0.88	0.86	0.82	High
6u3n/ranked_2	0.53	0.51	1.83	5.67	0.49	Medium	0.86	93.39	0.88	0.85	0.82	High
6u3n/ranked_3	0.60	0.56	1.67	4.48	0.45	Medium	0.86	93.63	0.88	0.85	0.82	High
6u3n/ranked_4	0.66	0.62	1.51	3.41	0.38	Medium	0.85	93.04	0.87	0.84	0.80	High
6xc9/ranked_0	0.58	0.56	1.67	5.16	0.40	Medium	0.87	93.65	0.89	0.86	0.84	High
6xc9/ranked_1	0.61	0.54	1.55	4.09	0.36	Medium	0.85	93.09	0.88	0.85	0.81	High
6xc9/ranked_2	0.67	0.65	1.42	3.98	0.35	Medium	0.85	93.09	0.88	0.85	0.82	High
6xc9/ranked_3	0.01	0.00	24.78	65.64	1.00	Incorrect	0.63	91.74	0.71	0.61	0.45	High
6xc9/ranked_4	0.31	0.26	2.79	9.29	0.46	Acceptable	0.45	86.07	0.56	0.42	0.25	High
6xco/ranked_0	0.88	0.89	0.67	2.52	0.16	High	0.90	95.57	0.92	0.90	0.88	High
6xco/ranked_1	0.91	0.91	0.58	2.23	0.12	High	0.90	95.44	0.91	0.90	0.88	High
6xco/ranked_2	0.91	0.94	0.64	2.33	0.17	High	0.90	95.02	0.91	0.90	0.88	High
6xco/ranked_3	0.03	0.02	17.45	30.62	0.97	Incorrect	0.54	89.00	0.63	0.51	0.29	High
6xco/ranked_4	0.05	0.00	14.86	22.59	1.00	Incorrect	0.46	89.22	0.58	0.44	0.20	High
6dfs/ranked_0	0.63	0.70	1.81	4.51	0.48	Medium	0.78	91.85	0.82	0.77	0.69	High
6dfs/ranked_1	0.56	0.49	1.89	4.39	0.55	Medium	0.65	90.39	0.72	0.64	0.47	High
6dfs/ranked_2	0.37	0.36	2.95	7.65	0.59	Acceptable	0.60	90.35	0.68	0.58	0.38	High

6dfs/ranked_3	0.52	0.45	1.94	5.15	0.33	Medium	0.51	87.78	0.62	0.49	0.24	High
6dfs/ranked_4	0.04	0.00	14.75	22.85	1.00	Incorrect	0.49	87.06	0.60	0.47	0.20	High
6dfw/ranked_0	0.66	0.63	1.42	3.97	0.31	Medium	0.79	91.92	0.82	0.78	0.69	High
6dfw/ranked_1	0.13	0.18	7.73	19.15	0.76	Incorrect	0.75	92.08	0.80	0.73	0.62	High
6dfw/ranked_2	0.11	0.09	6.78	16.95	0.87	Incorrect	0.69	91.30	0.76	0.68	0.53	High
6dfw/ranked_3	0.28	0.25	3.74	9.67	0.71	Acceptable	0.67	90.85	0.74	0.65	0.51	High
6dfw/ranked_4	0.18	0.18	5.26	13.16	0.76	Incorrect	0.64	90.60	0.71	0.62	0.46	High
6px6/ranked_0	0.60	0.61	1.85	4.25	0.36	Medium	0.87	93.77	0.89	0.86	0.84	High
6px6/ranked_1	0.79	0.75	1.05	2.34	0.25	Medium	0.86	93.74	0.88	0.85	0.81	High
6px6/ranked_2	0.75	0.73	1.21	2.69	0.27	Medium	0.85	93.74	0.88	0.85	0.81	High
6px6/ranked_3	0.12	0.01	6.88	12.74	0.94	Incorrect	0.49	87.82	0.61	0.47	0.20	High
6px6/ranked_4	0.15	0.07	6.06	12.05	0.77	Incorrect	0.48	86.40	0.60	0.45	0.20	High
7rdv/ranked_0	0.70	0.59	1.16	2.80	0.41	Medium	0.86	94.33	0.89	0.86	0.84	High
7rdv/ranked_1	0.72	0.61	1.15	2.79	0.40	Medium	0.86	94.51	0.89	0.86	0.84	High
7rdv/ranked_2	0.43	0.40	2.61	6.33	0.51	Acceptable	0.86	94.62	0.88	0.85	0.83	High
7rdv/ranked_3	0.50	0.50	2.29	5.77	0.33	Acceptable	0.86	94.35	0.88	0.85	0.83	High
7rdv/ranked_4	0.71	0.61	1.19	2.81	0.38	Medium	0.83	93.76	0.86	0.83	0.79	High
7sg0/ranked_0	0.64	0.59	1.49	3.89	0.46	Medium	0.88	93.84	0.90	0.88	0.86	High
7sg0/ranked_1	0.64	0.61	1.54	3.98	0.46	Medium	0.88	94.40	0.90	0.88	0.85	High
7sg0/ranked_2	0.64	0.64	1.56	4.22	0.45	Medium	0.88	93.87	0.90	0.87	0.85	High
7sg0/ranked_3	0.65	0.64	1.54	3.94	0.46	Medium	0.87	93.95	0.90	0.87	0.84	High
7sg0/ranked_4	0.61	0.61	1.67	4.55	0.45	Medium	0.86	93.63	0.88	0.85	0.82	High
7sg1/ranked_0	0.65	0.55	1.31	3.83	0.40	Medium	0.88	94.45	0.90	0.88	0.86	High
7sg1/ranked_1	0.36	0.38	3.39	8.16	0.42	Acceptable	0.85	93.38	0.88	0.85	0.82	High
7sg1/ranked_2	0.38	0.44	3.29	7.82	0.39	Acceptable	0.85	93.34	0.88	0.85	0.82	High
7sg1/ranked_3	0.36	0.38	3.30	8.00	0.45	Acceptable	0.84	92.88	0.87	0.83	0.80	High
7sg1/ranked_4	0.41	0.43	2.92	7.04	0.38	Acceptable	0.80	92.34	0.84	0.80	0.76	High
7z50/ranked_0	0.59	0.47	1.50	4.42	0.39	Medium	0.89	94.52	0.91	0.88	0.87	High
7z50/ranked_1	0.73	0.72	1.14	3.56	0.33	Medium	0.88	94.37	0.90	0.87	0.87	High

7z50/ranked_2	0.78	0.73	0.99	2.43	0.37	High	0.84	93.29	0.87	0.83	0.85	High
7z50/ranked_3	0.08	0.05	8.90	20.18	0.90	Incorrect	0.53	88.51	0.63	0.50	0.26	High
7z50/ranked_4	0.01	0.00	23.66	59.85	1.00	Incorrect	0.44	87.97	0.56	0.41	0.14	High
7t2c/ranked_0	0.76	0.69	1.07	2.19	0.29	Medium	0.89	92.54	0.90	0.88	0.86	Medium
7t2c/ranked_1	0.77	0.70	1.08	2.24	0.37	Medium	0.89	93.05	0.90	0.88	0.86	Medium
7t2c/ranked_2	0.77	0.71	1.04	2.36	0.30	Medium	0.88	92.31	0.90	0.87	0.85	High
7t2c/ranked_3	0.73	0.66	1.16	2.73	0.33	Medium	0.88	92.35	0.90	0.87	0.85	High
7t2c/ranked_4	0.54	0.62	2.00	6.25	0.51	Medium	0.85	91.90	0.88	0.85	0.84	Medium
7t2b/ranked_0	0.70	0.62	1.22	2.95	0.37	Medium	0.76	90.36	0.81	0.75	0.68	High
7t2b/ranked_1	0.54	0.52	2.14	4.82	0.48	Medium	0.76	89.75	0.80	0.75	0.66	High
7t2b/ranked_2	0.65	0.60	1.41	3.87	0.41	Medium	0.74	89.88	0.79	0.72	0.63	High
7t2b/ranked_3	0.59	0.53	1.76	4.06	0.45	Medium	0.72	89.56	0.77	0.70	0.59	High
7t2b/ranked_4	0.56	0.52	1.51	6.08	0.48	Medium	0.71	89.63	0.77	0.70	0.60	High

Docking quality metrics DockQ, Fnat, I-RMSD, L-RMSD, CAPRI, Fnonnat, and Peptide-MHC accuracy were calculated by the DockQ program.
[a]The ipTM score was modified to evaluate only the TCR-pMHC interface, instead of including all chains in the default ipTM score.
[b]We evaluated the peptide displacement in the MHC using DockQ with the “capri_peptide” setting, and report the CAPRI accuracy.

Table S4. Modeling success of AlphaFold, TCRmodel2, and TCRDock on a set of 20 recently released TCR-pMHC complexes.

PDB	MHC class	Vα %[a]	Vβ %[a]	AlphaFold 2.2[b]		AlphaFold 2.3[b]		TCRmodel2 (2.2)[b]		TCRmodel2 (2.3)[b]		TCRDock[c]	
				T1	T5	T1	T5	T1	T5	T1	T5	T1	T3
6zkw	1	89	92	0	2	1	2	1	3	3	3	1	1
7dzm	1	**88**	**88**	2	2	1	1	1	1	1	1	2	2
7ndq	1	89	91	2	2	2	2	2	2	2	2	0	1
7l1d	1	**89**	**89**	-1	-1	2	2	2	2	2	2	2	2
7rrg	1	88	92	0	1	2	2	0	2	2	2	0	1
7na5	1	**62**	**54**	0	1	1	1	1	1	0	0	1	1
7ow5	1	83	90	-1	-1	3	3	2	2	2	2	2	2
7qpj	1	**88**	**88**	3	3	2	3	3	3	3	3	2	2
7phr	1	87	94	2	3	3	3	3	3	3	3	2	2
7rk7	1	**88**	**85**	-1	-1	1	1	1	1	1	1	0	1
8d5q	1	66	93	2	2	2	2	2	2	2	2	2	2
8gvb	1	90	90	2	2	2	2	2	2	2	2	2	2
7n2n	1	91	93	-1	0	1	1	0	1	0	1	1	1
8gom	1	91	88	1	1	2	2	2	2	2	2	2	2
7rdv	2	64	92	-1	-1	3	3	2	2	2	3	2	2
7sg0	2	93	90	2	2	-1	2	2	2	2	2	1	1
7sg1	2	91	57	-1	2	1	2	2	2	2	2	2	2
7z50	2	**67**	**59**	-1	-1	-1	-1	2	3	3	3	3	3
7t2c	2	92	92	2	2	2	2	2	2	2	2	2	2
7t2b	2	91	94	2	2	2	2	2	2	2	2	1	1

All predictions were assessed by CAPRI criteria for Incorrect (denoted by "0"), Acceptable (denoted by "1"), Medium (denoted by "2"), and High (denoted by "3") modeling accuracy. Peptide displacement from the MHC was assessed using CAPRI criteria, and denoted with "-1". Cases without predictions generated were denoted with "-".

[a]Maximum α chain variable domain (Vα) and β chain variable domain (Vβ) sequence identity with any TCR from known TCR-pMHC structures of the same class from on or before the 2021-09-30 date cutoff. Values in bold denote complexes with both chains below a 90% sequence identity level, indicative of structures with limited germline and/or CDR3 sequence match to previously described known structures for both chains.

[b]Five predictions per case were generated, and predictions were ranked by model confidence score. A template date cutoff of 2021-09-30 was applied to disable the use of recently released structures (overlapping with the benchmark set) as templates. T1 indicates accuracy for top model, and T5 indicates best accuracy among all five models.

[c]TCRDock generated three predictions per case, and predictions were ranked by PAE score. T1 indicates accuracy for top model, and T3 indicates best accuracy among all three models.

Table S5. Receiver operating characteristic area under the curve (AUC) values for CAPRI accuracy classes as a function of model confidence metrics for TCRmodel2 predictions on the benchmarking set of 48 cases.

Score	Incorrect vs. Acceptable, Medium, High	Incorrect, Acceptable vs. Medium, High	Incorrect vs. Medium, High
Model confidence	0.91	0.91	0.97
pTM	0.91	0.91	0.97
ipTM	0.91	0.91	0.97
TCR-pMHC ipTM[a]	0.91	0.91	0.97

AUC values were calculated using the pROC package in R (1).
[a]ipTM score considering only the TCR-pMHC interface, rather than the interfaces between all chains as used by the default ipTM score.

Table S6. CDR loop modeling accuracy of TCRmodel, AlphaFold, and TCRmodel2 (2.3 AlphaFold model) for unbound TCR structures.

PDB	MHC class	TCRmodel						AlphaFold 2.2						AlphaFold 2.3						TCRmodel2 (2.3)					
		**1α**	**2α**	**3α**	**1β**	**2β**	**3β**	**1α**	**2α**	**3α**	**1β**	**2β**	**3β**	**1α**	**2α**	**3α**	**1β**	**2β**	**3β**	**1α**	**2α**	**3α**	**1β**	**2β**	**3β**
6zkw	1	0.88	0.77	2.82	0.69	0.87	3.84	0.69	0.44	1.70	0.53	0.44	3.60	0.54	0.50	1.69	0.47	0.41	3.89	0.39	0.40	2.40	0.40	0.35	3.71
7na5	1	1.81	0.98	2.06	0.79	1.22	4.48	0.74	0.39	0.65	0.65	0.81	1.38	0.67	0.40	0.55	0.60	0.96	1.33	0.76	0.37	0.69	0.66	0.76	1.53
7ow5	1	0.78	0.99	3.73	0.64	0.55	2.96	0.52	1.49	1.29	0.46	0.46	1.22	0.55	1.17	1.17	0.39	0.41	1.27	0.32	0.61	1.08	0.32	0.36	1.40
7qpj	1	0.76	0.74	2.82	0.55	0.42	2.56	0.47	0.42	0.41	0.46	0.38	1.02	0.37	0.41	0.53	0.41	0.45	0.57	0.42	0.48	0.56	0.31	0.33	0.85
8gvb	1	2.59	0.96	2.78	1.25	1.41	3.23	2.19	0.86	2.71	0.94	1.46	0.94	2.02	0.87	2.44	0.95	1.32	1.37	1.79	0.84	2.02	0.60	0.60	1.17
7phr	1	2.66	1.08	3.06	0.67	0.81	1.21	0.50	0.55	0.69	0.63	0.97	1.12	0.52	0.55	0.78	0.63	0.90	1.17	0.53	0.51	0.71	0.38	0.66	1.16
7ndq	1	1.81	0.93	4.21	1.27	1.21	2.19	0.62	1.20	0.94	0.75	1.33	2.51	0.70	1.25	1.57	0.88	1.23	2.78	0.64	0.67	3.17	0.35	1.36	2.95
7rrg	1	0.97	0.67	4.60	0.66	0.58	3.71	1.03	1.25	4.85	0.45	0.47	3.09	1.11	1.16	4.44	0.40	0.52	3.33	0.62	0.44	4.65	0.35	0.57	1.86
7l1d	1	2.23	0.91	6.16	0.87	0.49	7.68	2.21	0.63	4.94	0.54	0.73	10.05	2.39	0.69	5.00	0.80	0.66	8.40	2.32	0.73	5.05	0.62	0.57	9.65
7dzm	1	2.28	0.92	5.12	0.80	1.03	1.52	0.50	0.41	4.03	0.74	1.23	1.31	1.69	0.41	2.51	0.75	1.27	1.00	0.30	0.38	1.00	0.70	1.17	1.13
7sg1	2	1.73	0.68	5.34	1.39	1.82	1.97	1.63	1.25	1.63	0.62	0.82	0.62	0.89	1.19	0.81	0.88	1.10	2.00	0.74	0.55	1.02	0.62	0.78	1.78
7rdv	2	0.63	1.43	4.50	0.49	0.48	1.31	0.46	0.73	1.25	0.27	0.27	0.49	0.60	0.86	1.94	0.34	0.23	2.57	0.39	0.71	1.25	0.26	0.27	0.44
7z50	2	0.69	0.85	2.73	0.99	1.04	2.39	0.61	0.73	2.70	0.46	1.28	2.44	0.64	0.74	2.74	0.43	1.27	2.54	0.51	0.75	2.41	0.40	1.03	2.34

Modeling accuracy is shown as backbone root-mean-square distance (RMSD) in Ångstroms of the respective CDR loop between model and native structures, after superposition of framework regions.

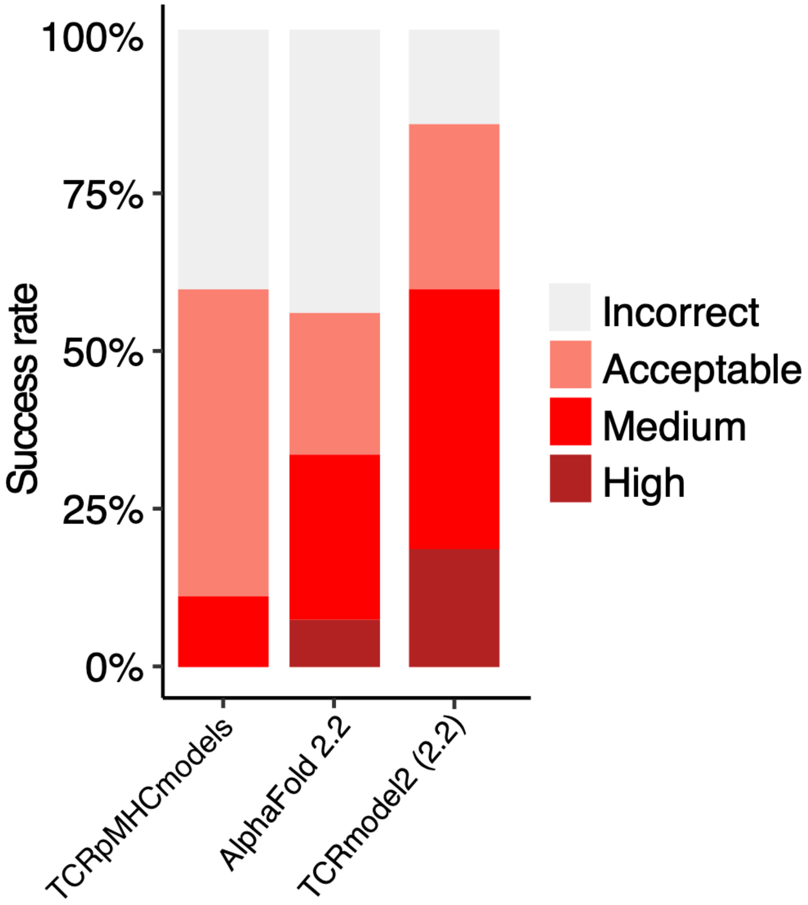

Figure S1. Modeling success comparison of TCRpMHCmodels, AlphaFold 2.2, and TCRmodel2 (with the AlphaFold 2.2 model), on the subset of 27 Class I TCR-pMHC benchmarking cases that were modeled by TCRpMHCmodels (from **Table S1**). For AlphaFold and TCRmodel2, 5 predictions were generated per case, and the top-ranked model was used for success calculation. All models were assessed by CAPRI criteria of Incorrect, Acceptable, Medium and High accuracy.

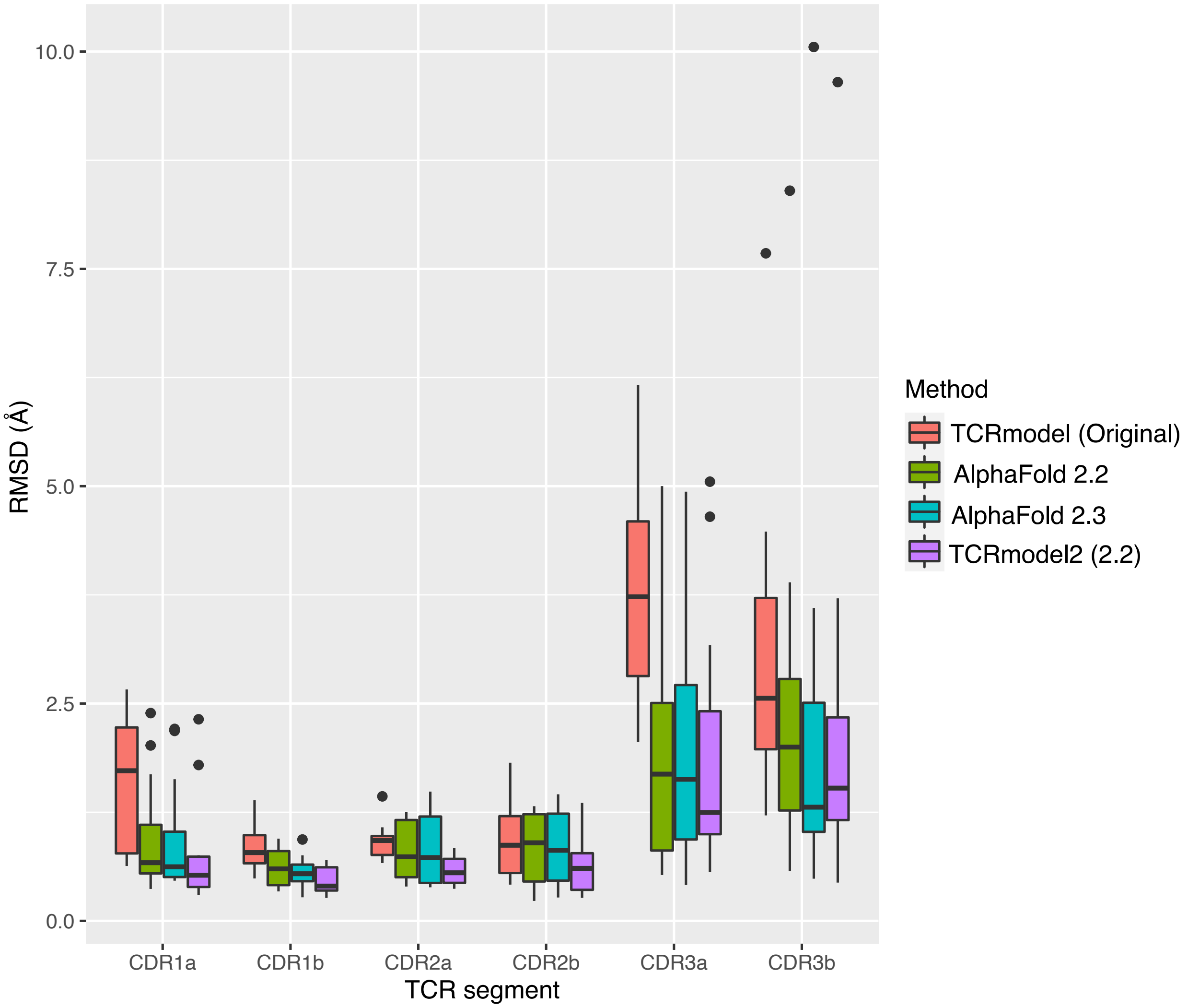

Figure S2. CDR loop modeling accuracy for unbound TCR models. Backbone root-mean-square distances (RMSDs) between experimentally determined structure and model were calculated for each CDR, after superposition of framework region of the variable domain. Test cases correspond to 13 recently released nonredundant TCR structures which are a subset of our recent TCR-pMHC benchmark cases for which a model was successfully generated by the original TCRmodel (cases and individual RMSDs are shown in **Table S3**). Methods compared are TCRmodel (original), AlphaFold 2.2, AlphaFold 2.3, and TCRmodel2 (with AlphaFold 2.3 model).

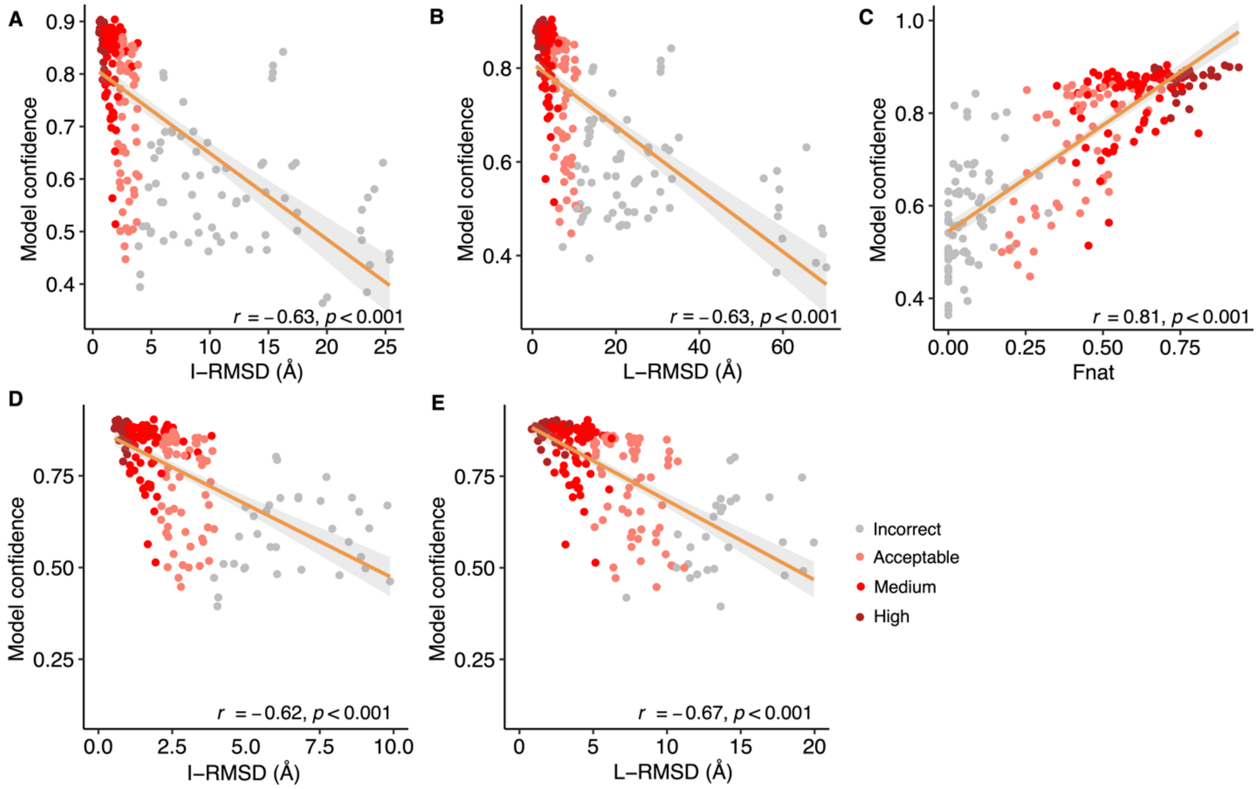

Figure S3. Comparison of model confidence score and model accuracy criteria. Model confidence of all 5 models for 48 cases generated by TCRmodel2 are shown in comparison with model accuracy with respect to the experimentally determined structure, with each model represented as a point and colored by CAPRI accuracy level. Pearson's correlation coefficient and the associated p-value are noted on the lower right corner, and the orange line represents the linear fit (with 95% confidence area in gray). Comparisons of model confidence score versus (A) I-RMSD, (B) L-RMSD, and (C) Fnat are shown. Additionally, comparisons of model confidence score versus (D) I-RMSD with values less than or equal to 10 Å, and (E) L-RMSD values less than or equal to 20 Å are shown.

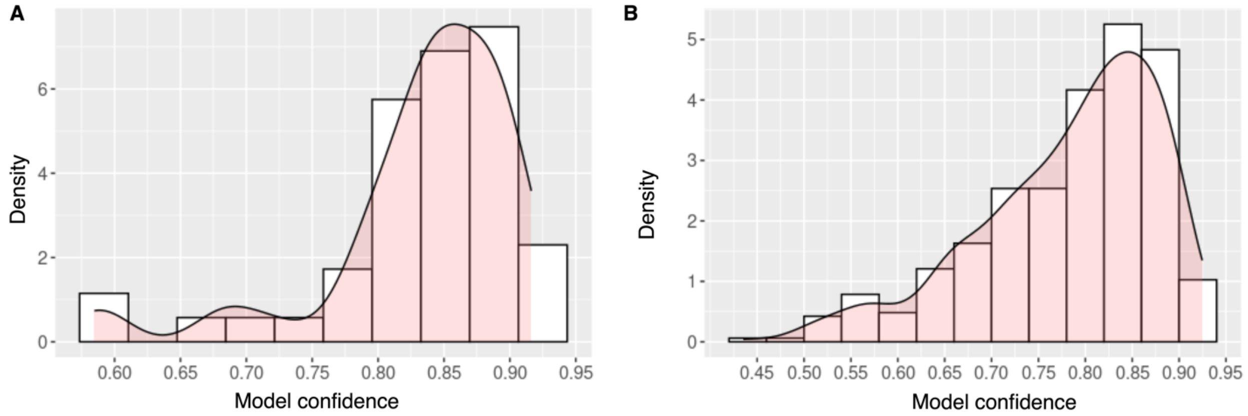

Figure S4. Model confidence distribution for TCR-pMHC sequences. A dataset of TCR-pMHC sequences was collected from the VDJdb database (2), consisting of 414 Class I TCR-pMHC and 47 Class II TCR-pMHC sequences that have no redundancy with TCRs from known TCR-pMHC complex structures (cutoff date 9/30/2021) and each other, based on our nonredundancy criteria used for benchmark assembly. Only samples with a VDJdb confidence score >=1 were considered. We then modeled all samples using our TCRmodel2 and obtained the model confidence of the top-ranked model for each complex. The distributions of model confidence scores are shown for (A) Class I TCR-pMHC complexes and (B) Class II TCR-pMHC complexes. Plots were generated using the ggplot2 package (3) in R (r-project.org), with a bin width of 0.04.

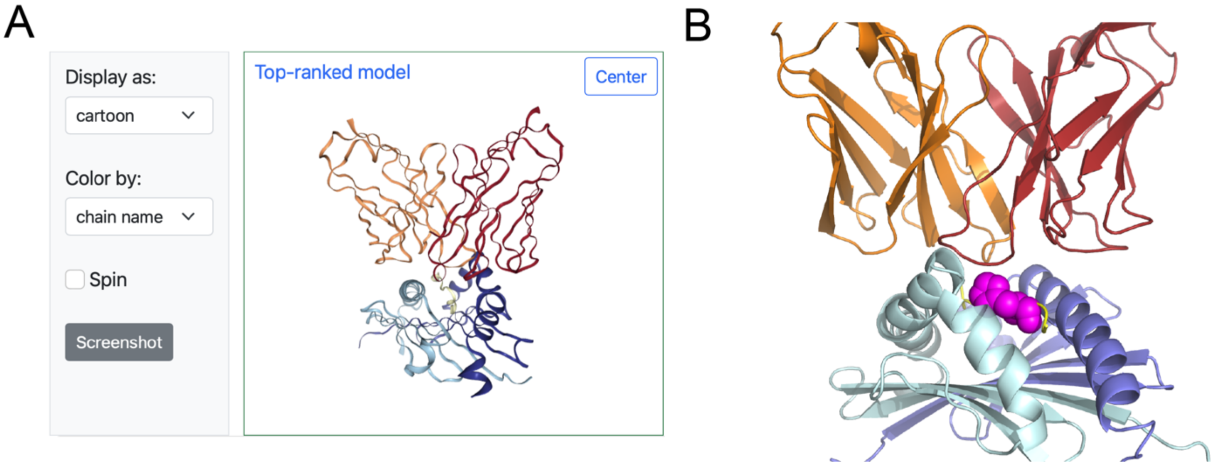

Figure S5. Example Class II TCR-pMHC modeling output from TCRmodel2. (A) A previously identified neoantigen-specific TCR (4) was modeled in complex with its target, a p53 R175H neoantigen and Class II HLA-DRB1*13:01 MHC, and the visualized top-ranked model from the TCRmodel2 results page is shown. The model is shown in cartoon representation with TCR α chain (red), TCR β chain (orange), peptide (yellow), MHC α chain (cyan), and MHC β chain (blue) colored separately. (B) The PDB file for the top-ranked model, downloaded from the TCRmodel2 results page, is shown visualized in PyMOL (Schrödinger, Inc.). Chains are colored as in (A), and the neoantigen mutant residue (H175) is shown in sphere representation and colored magenta.

Supplemental References

1. Robin, X., Turck, N., Hainard, A., Tiberti, N., Lisacek, F., Sanchez, J.C. and Muller, M. (2011) pROC: an open-source package for R and S+ to analyze and compare ROC curves. *BMC bioinformatics*, **12**, 77.
2. Bagaev, D.V., Vroomans, R.M.A., Samir, J., Stervbo, U., Rius, C., Dolton, G., Greenshields-Watson, A., Attaf, M., Egorov, E.S., Zvyagin, I.V. *et al.* (2020) VDJdb in 2019: database extension, new analysis infrastructure and a T-cell receptor motif compendium. *Nucleic Acids Res*, **48**, D1057-D1062.
3. Wickham, H. (2016) *ggplot2: Elegant Graphics for Data Analysis*. Springer-Verlag New York.
4. Malekzadeh, P., Pasetto, A., Robbins, P.F., Parkhurst, M.R., Paria, B.C., Jia, L., Gartner, J.J., Hill, V., Yu, Z., Restifo, N.P. *et al.* (2019) Neoantigen screening identifies broad TP53 mutant immunogenicity in patients with epithelial cancers. *J Clin Invest*, **129**, 1109-1114.
